# Risk Factors for Anaplastic Thyroid Carcinoma: A Case Series From a Tertiary Referral Center for Thyroid Surgery and Literature Analysis

**DOI:** 10.3389/fonc.2022.948033

**Published:** 2022-07-07

**Authors:** Giuseppa Graceffa, Giuseppe Salamone, Silvia Contino, Federica Saputo, Alessandro Corigliano, Giuseppina Melfa, Maria Pia Proclamà, Pierina Richiusa, Sergio Mazzola, Roberta Tutino, Giuseppina Orlando, Gregorio Scerrino

**Affiliations:** ^1^ Department of Surgical, Oncological and Oral Sciences, University of Palermo, Palermo, Italy; ^2^ Department of Surgical Oncological and Oral Sciences, Unit of General and Emergency Surgery, University of Palermo, Palermo, Italy; ^3^ Section of Endocrinology-Department of Health Promotion Sciences Maternal and Infantile Care, Internal Medicine and Medical Specialties (PROMISE), University of Palermo, Palermo, Italy; ^4^ Unit of Clinical Epidemiology and Tumor Registry, Department of Laboratory Diagnostics, Policlinico “P. Giaccone” University of Palermo, Palermo, Italy; ^5^ Department of General and Specialized Surgery, City of Health and Science Hospital of Turin, Turin, Italy

**Keywords:** anaplastic thyroid carcinoma, risk factors, multinodular goiter, thyroidectomy, prognosis

## Abstract

Anaplastic thyroid carcinoma (ATC) is a very rare and extremely aggressive disease with a very poor prognosis. Several risk factors have been hypothesized, but there is no clear-cut literature data on it. We reviewed the literature concerning risk factors for ATC and analyzed the institutional database from 2005 to 2022. In total, 15 papers were suitable for review, while the retrospective data collection search, conducted on our institutional database, provided 13 results. In our experience, in agreement with literature data, ATC seems to be a neoplasm peculiar to old age (in our database, mean age is 72 years), with a higher prevalence in subjects with a low level of education and a long history of multinodular goiter (MNG). The role of cigarette smoking and blood group, hypothesized on some literature data, was more uncertain, although the small sample size evaluated probably had a great influence on these results. The higher incidence of the disease in individuals with a history of MNG could suggest more aggressive choices in the treatment of a benign disease, in contrast to current practice. However, this may be a highly questionable point considering that ATC accounts for no more than 2% of all thyroid neoplasms in surgical departments, even those dedicated to endocrine neck surgery. Further studies are therefore necessary for a step forward in this direction.

## Introduction

Anaplastic thyroid carcinoma (ATC) is a very rare and extremely aggressive disease with a very poor prognosis.

It accounts for 2%–3% of all thyroid neoplasms. It is composed of undifferentiated thyroid follicular cells, which require immunohistochemical and ultrastructural support to determine their epithelial origin. Beyond significant local invasion, ATC often presents with local and distant lymph node metastatic spread (1).

In Europe, the estimated incidence, during the years 2000–2007, was 0.1/100,000 subjects per year (2).

It is more frequent in the elderly, with only 25% of patients under 60 years of age and more than 90% are over 50 years old, mostly in their 70s and 80s.

ATC has a higher prevalence in women, with an estimated female-to-male (F:M) ratio of 1.5–3:1. (3, 4)

This cancer may arise *de novo* or in patients with a long history of multinodular goiter (MNG) or may represent the evolution of an unrecognized differentiated carcinoma (20%–25% of cases) (5).

At the time of diagnosis, about 80% of patients have locally advanced disease and almost 50% already have distant metastases (lung, bone, brain). The median survival from diagnosis is 5 months, and less than 20% of patients survive 1 year (6).

The literature shows that more than one-third of patients with ATC have a history of goiter before diagnosis (median duration, 8.5 years) (3, 7) and that this cancer is twice as common in areas with endemic goiter. Over the last few years, there has been a reduction in the incidence of ATC: in an effort to provide a possible explanation for this finding, it seems logical to relate this decline to iodine prophylaxis, which would act in the same direction by reducing the incidence of benign goitrogenic disease and, concurrently, of ATC (8–12).

Insofar that a specific risk context for this neoplasm is not yet well-defined, in reviewing our case series, we asked ourselves:

- Given that only 20%–25% represent the evolution of a differentiated thyroid carcinoma (DTC), are the risk factors the same as for a DTC, and more importantly, what other risk factors determine the evolution of a DTC into ATC?
- Is it possible to identify ATC-specific risk factors in order to achieve early diagnosis in patients suffering from MNG?
- Furthermore, in the era of increasing conservatism in thyroid surgery, including neoplastic disease, what is the actual risk of anaplastic carcinoma?

Clarifying risk factors could make disease prevention possible through early treatment of precursor conditions.

We reviewed the literature on specific risk factors for ATC and analyzed risk factors of ATC cases diagnosed by cytology and/or histology occurring in the surgical department of our university hospital from 2005 to 2022. These data were obtained from the pathology database of our institution and compared with literature. The intermediate endpoint of the study was to establish, on the basis of literature data and our institution’s experience, potential risk factors for ATC diagnosed by histology. The final endpoint was to assess whether there are risk criteria that would change the current indications for thyroid surgery in defined risk settings.

## Materials and Methods

We searched for specific studies on risk factors related/correlated to ATC, including all types of English language articles in PubMed, Web of Science, and Scopus articles published from 2000 to 2022.

The following MeSH terms were adopted: “anaplastic thyroid cancer risk factors” or “anaplastic thyroid carcinoma risk factors.” The search resulted in 139 and 108 articles, respectively. Once duplicate papers were excluded, the abstracts were read, and the unsuitable papers were excluded. We included only papers whose topic was specifically addressed on risk factors for anaplastic carcinoma, excluding papers on risk factors for benign and malignant nodular disease in general.

**Figure 1:**
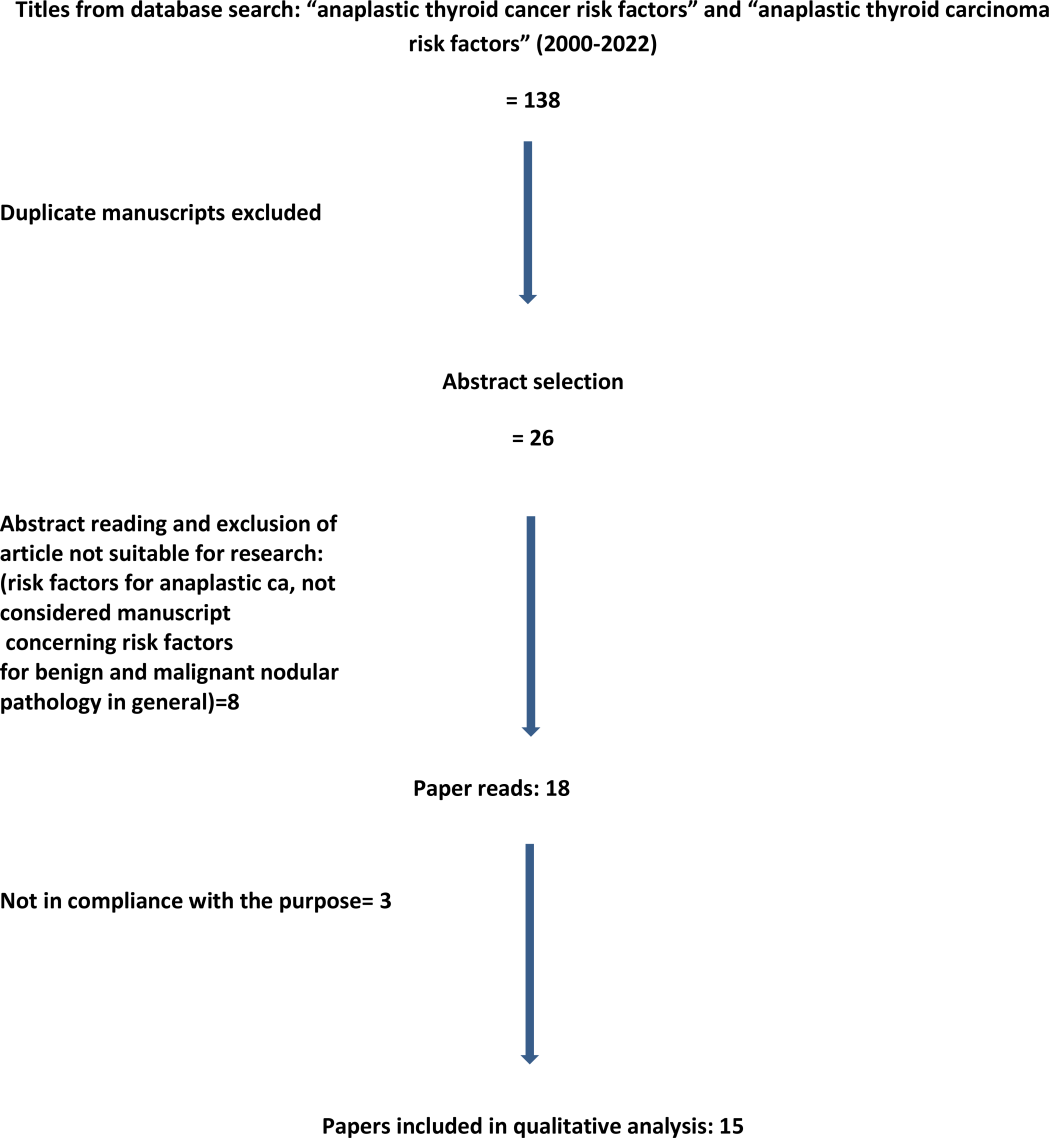
Prisma diagram detailing the literature search process and article selection.

After this literature review, the number of articles considered eligible was only 15 (**Figure 1**).

After extrapolating from the literature review the risk factors recurrent in the enrolled studies, we have assessed the variables most frequently found in the literature: demographic data (sex, age, level of education) and smoking status. In addition, variables such as history of thyroid disease and possible suppressive therapy with L-T4, personal history, such as history of head and neck irradiation, therapy with radioiodine-131, diabetes mellitus (DM), neoplasms in other sites, body mass index (BMI), blood group, and hormonal history in women were evaluated.

In this study, we aimed to perform a retrospective assessment of potential risk factors for ATC in a group of patients with definite histologic diagnosis.

This study examines 13 cases of patients with ATC that occurred in the surgical department of Policlinico Universitario “P. Giaccone” of Palermo, a third-level center for thyroid surgery, from 2005 to 2022. By recruiting patients from surgical databases, we obtained a group of patients, albeit limited, but with a definite diagnosis.

Patients with a plausible diagnosis were excluded, even if strongly suspected from a clinical point of view, in the absence of a histological diagnosis. Therefore, patients were selected on the basis of a double search, one based on the databases of the General and Emergency Surgery and Oncological Surgery units of the Policlinico Universitario “P. Giaccone” of Palermo and the other on the database of the Surgical Pathology Service of the same institution. Therefore, we excluded patients who, although registered as ATC or other similar terms, were not part of the databases of the abovementioned services.

We also excluded cases of advanced papillary and follicular carcinomas, poorly differentiated tumors, and insular carcinomas.

In the present study, we aimed to analyze the data available at our institution on potential risk factors for ATC and to compare them with literature data. The surgery performed on each individual patient, the adjuvant therapy, and the outcome are summarized in **Table 1**. Given the small sample of patients enrolled, the statistical evaluation was only descriptive.

**Table 1:** Pt, patient; TT, total thyroidectomy; CND, central neck dissection; LND, lateral neck dissection; TS, tracheal stent; CT, chemotherapy; RT, Radiotherapy.

Pt	Age	Sex	Surgery	Chemotherapy/Radiotherapy (CT/RT)	Survival (months)
1.	71	F	TT + CND	None	20
2.	83	M	TT	CT	24
3.	82	F	None	None	1,5
4.	77	F	TT + CND + right LND + tracheostomy	CT	4
5.	77	M	None	CT	3
6.	36	F	TT + CND	Unknown	Lost at follow-up
7.	61	F	Biopsy	RT	3
8.	81	F	Debulking	CT + RT	6
9.	75	M	TT + LND	CT	40
10.	67	F	Biopsy	CT	2
11.	76	M	Biopsy + TS	None	1
12.	75	F	Biopsy + TS	None	1
13.	79	F	Biopsy + TS	None	2

## Results

### Demographic Data

Analysis shows that the majority of our ATC patients are women, with an F:M ratio of 2.25:1 (9 women and 4 men).

Almost all were over 60 years old, with a mean age of 72 years (range: 36–83) and a median age of 76 years. Only one patient was 36 years old at the time of diagnosis. She was a young pregnant woman at the 11th week of gestation when, due to the recent appearance of a painful swelling in the neck associated with dysphagia, she underwent an ultrasound examination and a nodule was found in the left lobe of the thyroid gland, Bethesda 6 on Fine Needle Aspiration Biopsy (FNAB). She underwent total thyroidectomy and lymphadenectomy of the left central hemi-compartment at the end of pregnancy, including a histological diagnosis of anaplastic carcinoma of the left lobe of the thyroid (transverse diameter maximum 3.1 cm) with anaplastic microfocus in the right lobe (1.9 × 1.2 mm) and 1/9 lymph nodes as site of metastasis.

**Figure 2:**
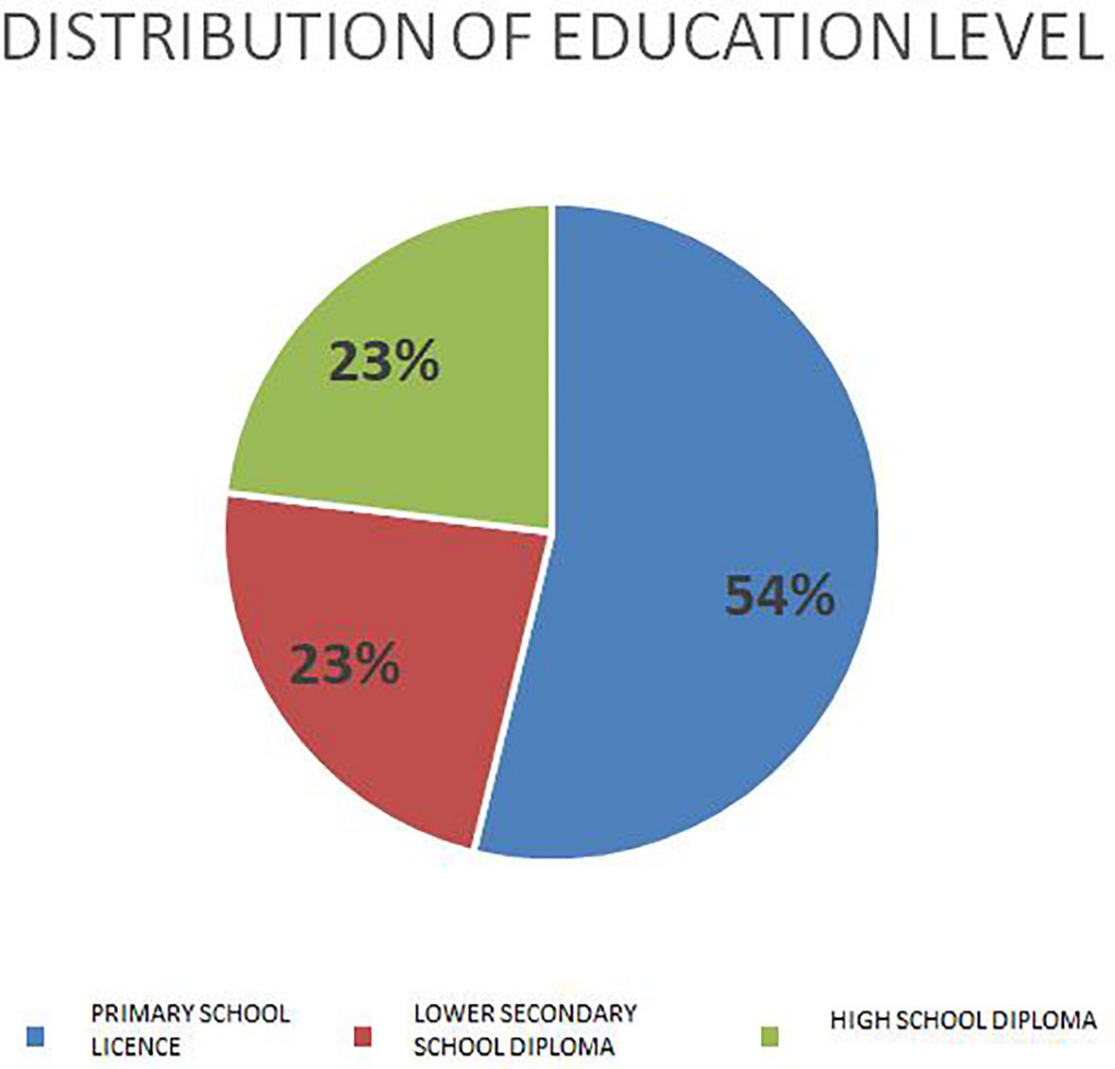
Level of education.

Regarding the level of education, most had a low level of education: 54% had only a primary school certificate or no educational degree, 23% had a lower secondary school diploma, and only 23% had a high school diploma; no one had a college or university degree (**Figure 2**).

Concerning the smoking habit, out of 13 patients, 5 were ex-smokers, 2 were smokers, and 6 had never smoked.

- History of thyroid disease/MNG/levothyroxine suppressive therapy

Almost all of the patients had a history of known thyroid nodular disease.

Of 13 patients, 10 (76.9%) had a history of long-standing MNG (>8 years).

- Personal medical history: history of head and neck irradiation, therapy with Radium Iodine-131, DM, neoplasms in other locations

Only one female patient had a history of DM (7.7%). One male patient had a history of head and neck irradiation; one patient had a history of thyrotoxicosis treated with Radium Iodine-131.

Three patients had a personal history of malignancy in other sites (one with previous prostate cancer, one with a history of uterine malignancy, and another one had a colic stromal tumor treated 4 years previously).

- BMI, blood group, and hormonal history in women

**Figure 3:**
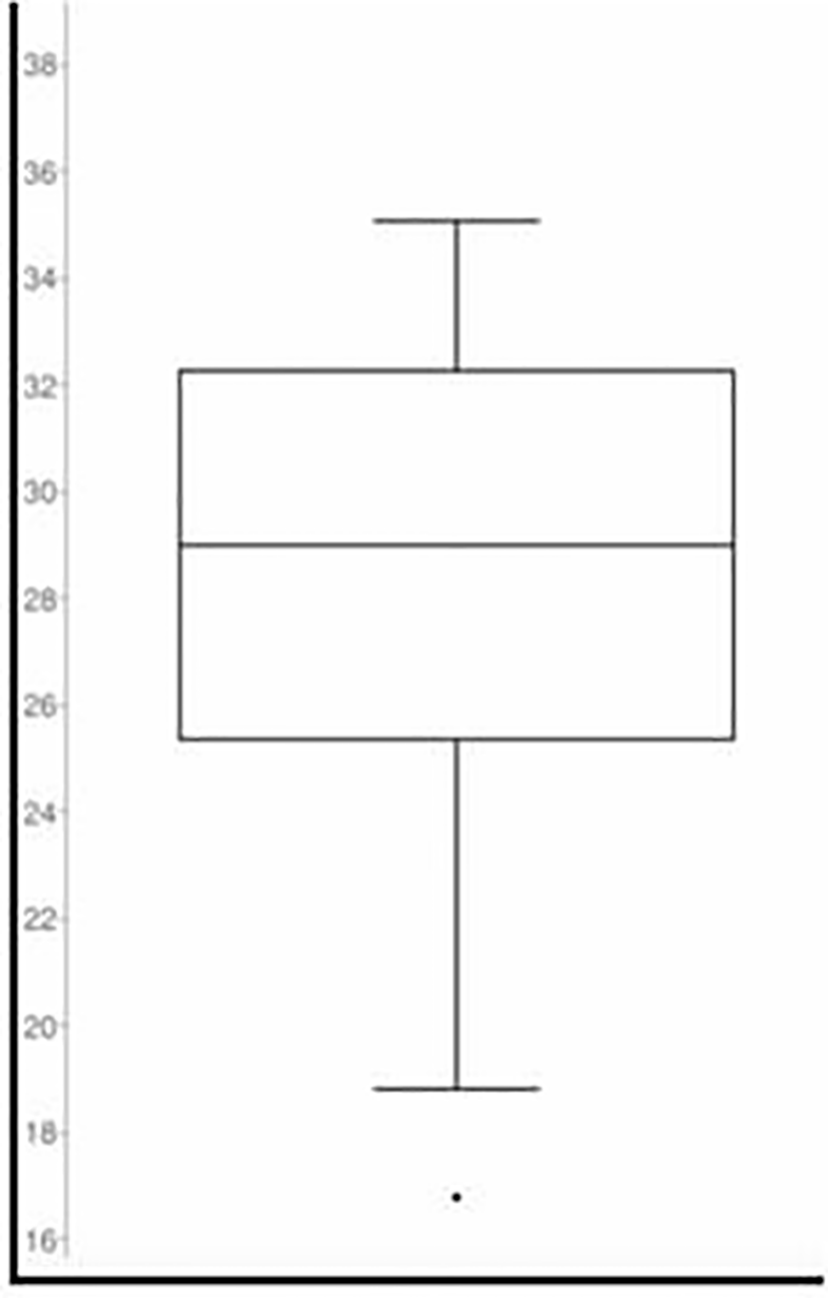
BMI grouping in Box Plot.

The mean BMI of patients with ATC was 28.2 with a range from 16.8 to 39.9 and a median of 29 (**Figure 3**).

We observed a history of recent major weight loss in two female patients with BMI of 16.8 and 18.8.

**Figure 4:**
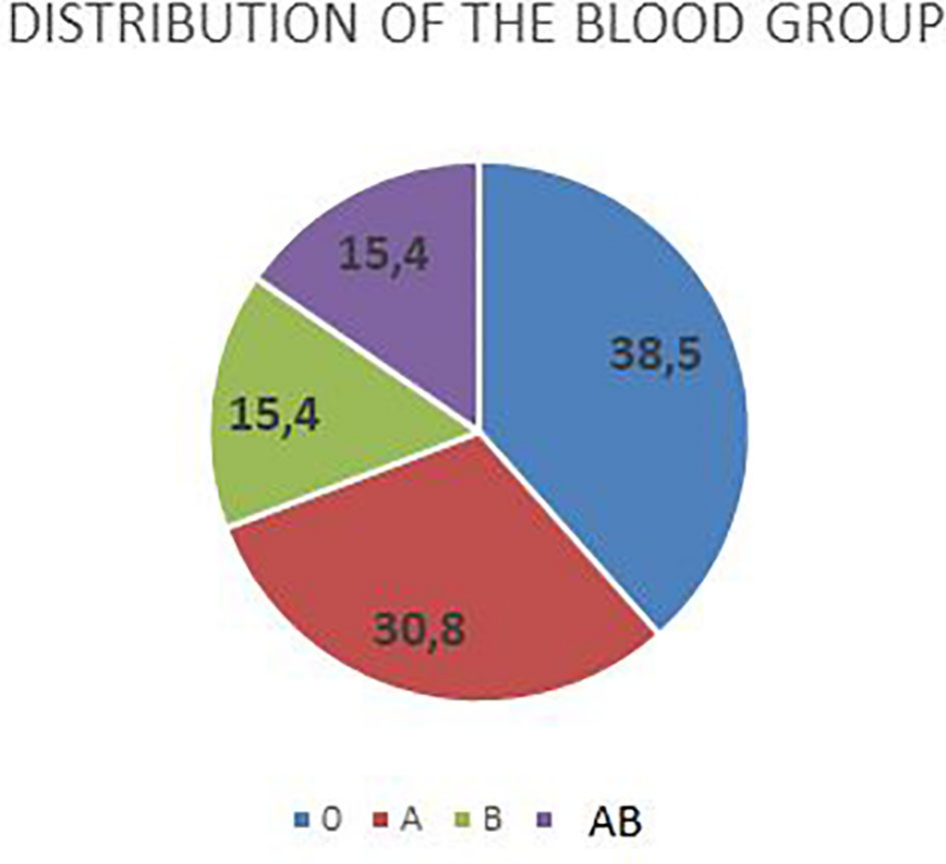
Blood group.

Concerning the blood group, 4 patients had blood group A+, 5 patients had blood group 0 (1 0 RH negative and 4 0 RH positive), 2 had blood group B+, and 2 AB (**Figure 4**).

Regarding the hormonal history in women, the mean age of menarche = 12.8 years (range 10–16 years).

The mean age of menopause = 53.4 years (range 50–57 years).

The demographic data of the institutional case series are summarized in **Table 2**.

**Table 2:** Demographic data.

Variable	Men, N (%)	Women, N (%)	Total (%)
Sex	4 (30.8%)	9 (69.2%)	13 (100%)
Age (median)	72.3 (76)		
BMI (median)	28.2 (29)		
Diabetes	Yes, N (%)	No, N (%)	Total (%)
	1 (7.7%)	12 (92.3%)	13 (100%)
Goiter	Yes, N (%)	No, N (%)	Total (%)
	10 (76.9%)	3 (23.1%)	13 (100%)
Neck irradiation	Yes, N (%)	No, N (%)	Total (%)
	1 (7.7%)	12 (92.3%)	13 (100%)
History of cancer	Yes, N (%)	No, N (%)	Total (%)
(other than thyroid)	3 (23.1%)	10 (76.9%)	13 (100%)
Menarche	Mean age (Median)		
	12.8 (13)		
Menopause	Mean age (Median)		
	53.4 (54)		
L-T4 treatment	Yes, N (%)	No, N (%)	Total (%)
	3 (23.1%)	10 (76.9%)	13 (100%)

## Discussion

In the literature review, the first case–control study on ATC risk factors is a Serbian study that started in 2004 and continued 3 and 4 and 10 years later.

The first part of the study (started/published in 2004) involved a smaller number of cases and a control group represented by patients recruited at the hospital for non-autoimmune rheumatic diseases. According to this study, independent risk factors for ATC were a clinical history of goiter, living in a goiter-endemic area, a personal history of other non-thyroid neoplasms, DM, and a low level of education (13).

The second part of the study (started/published in 2007) involved a larger number of cases and a control group of goiter patients in order to identify any independent risk factors. The only significant difference between cases and controls was a longer history of MNG in ATC cases (>10 years). According to the results obtained in this Serbian study, with patients with goiter as control, anaplastic cancer is associated with a lower level of education, a personal history of other neoplastic diseases, blood group B, late menarche, and early first full-term pregnancy. These two reproductive factors (late menarche and early first full-term pregnancy) were found to be statistically significant as risk factors associated with ATC but cannot be considered as independent risk factors, as they are generally risk factors for thyroid cancer, the incidence of which is several times higher in women than that in men (14–16).

In the case of the control group represented by patients with papillary carcinoma, no statistically significant independent risk factors for ATC were found except for the age factor (17).

In 2014, a new study was conducted in which the results of the previous studies were briefly summarized, and new results were presented, including a control group based on the general population with the same residence of the cases. This is the only long-standing case–control study aimed to identify potential risk factors for the development of ATC found on PubMed. The results show that the majority of patients with ATC are women with an F:M ratio of 1.5:1, with a mean age of 67 years (37–88 years). Most of them are in the seventh decade of life (51.2%). In addition, 69.8% lived in urban areas. There were no significant differences in habits such as cigarette, alcohol, and coffee consumption. Living in a goiter-endemic area was not statistically significant. The study identified three independent risk factors for ATC compared with previous studies in which five risk factors were identified, thus excluding goiter because the control group in the 2007 study consisted of patients with goiter and excluding MNG-endemic area because the control group in this study was composed of patients living in the same areas. The three independent risk factors for ATC identified by this study are diabetes, a personal history of other non-thyroidal malignancies, and type B blood group, as well as a low level of education. The search for independent risk factors for ATC is intended to reduce bias; however, one of the major problems with these studies is that they consist of a relatively small size of the group with ATC (18).

However, these risk factors are too general, given the epidemiology of DM in the western world and the increased incidence of malignancy with aging. There are no other data in the literature correlating anaplastic thyroid cancer with blood group, although there are data on other cancers at other sites.

Some older studies have correlated ATC with I131 and L-thyroxine therapy.

The study by Zivaljevic et al. (18) does not demonstrate this association (only one patient was previously operated for well-differentiated thyroid cancer with subsequent therapy with 131I Radioiodine before anaplastic transformation occurred; five patients in the case group were on L-thyroxine replacement or suppressive therapy).

A background of MNG and a history of radioactive iodine therapy is not uncommon (19), although these risk factors cannot be identified as independent.

ATC most frequently affects the elderly with a long history of MNG, reported in up to 50% of cases, and in 20% of cases, patients have a history of DTC, usually papillary type (20, 21).

Chandrakanth and Shaha (22), in a 2006 review, demonstrated that previous or concomitant thyroid disorder (benign or DTC) is a risk factor for the development of ATC (8, 10, 23–29) and that ATC is twice as common in goiter-endemic areas and that the decline in recent years may be due to iodine prophylaxis (8–12) and improvements in socioeconomic status, which have been shown to be associated with a reduction in the incidence of ATC (11). This study shows that the incidence of ATC is higher in goiter-endemic areas and in patients with previous papillary or follicular cancer who were not adequately treated (8, 26, 30–33). This has led to the suggestion that aggressive oncological treatment may be responsible for reducing the incidence of ATC by eliminating its transformation potential (8).

The coexistence of DTC and ATC is very well documented (31, 34, 35). Indeed, some studies have reported DTC to ATC transition zones in the same sample and even findings of tiny DTC foci within ATC and *vice versa* (36, 37).

Although any DTC type can be found in association with ATC, papillary cancer is the most common (8, 10, 26, 28, 32, 38). Within papillary cancer, the biologically more aggressive forms (insular and high cell type) are most commonly found associated with ATC, reinforcing the theory of neoplastic transformation.

The pathogenesis of de-differentiation from papillary carcinoma to ATC has been attributed, in some cases, to the acquisition of additional genetic changes including mutation in the p53 oncosuppressor gene (21). Increased expression of the urokinase-type plasminogen activator (uPA) system has been reported to be associated with tumor invasion, neo-angiogenesis, and metastatic spread in thyroid cancers, and Aurora kinases are associated with severe mitotic abnormalities. Based on this evidence, uPA and Aurora kinases are considered potential therapeutic targets for the treatment of ATC (38, 39).

In some cases, it has been suggested that radiotherapy of differentiated tumor (DTC) may play a role in the transformation to undifferentiated tumor (20, 40).

Maatouk et al. (19) described a case report of a 90-year-old patient with a long history of MNG (>45 years) and several bouts of thyrotoxicosis treated with I131 radiotherapy and subsequent diagnosis of advanced ATC that caused his immediate death (2 days after diagnosis), suggesting that a long history of MNG and radioiodine therapy are not uncommonly associated with ATC.

In the few cases where ATC occurred after radioactive iodine treatment for papillary carcinoma, p53 abnormalities were documented in both primary tumor and ATC by immunohistochemical evaluation (21).

After these studies, the issue of radioactive iodine as a cause of further induced p53 mutation was raised (questioned).

Leitzmann et al. (41) in a 2010 US prospective study analyzed the relationship between BMI/adiposity and physical activity and thyroid cancer with evidence of a positive relationship between increased BMI and thyroid cancer, particularly papillary, follicular and anaplastic, non-medullary thyroid cancer. This association is greater in men than that in women. No significant relationship between physical activity and DTC was demonstrated, although there was some evidence of a positive relationship with the anaplastic cancer (41).

Engeland et al. (42) in a Norwegian study showed a stronger association between BMI and follicular carcinoma and anaplastic carcinoma than papillary thyroid cancer.

This hypothesis is supported by a recent study suggesting that an obesity-inducing high-fat diet may play an important role in thyroid carcinogenesis. This study, using a ThrbPV/PV/Pten+/- mouse model, points out that such a diet can cause increased proliferation of thyroid cancer cells, induced anaplastic changes, and increased serum leptin levels (43) through activation of the Janus kinase 2/signal transducer and activator of transcription (3 JAK2-STAT3) signaling pathway. Activation of Janus kinase 2/signal transducer and activator of transcription (3 JAK2-STAT3) by other cytokines such as Interleukin 6 (IL-6) has also been reported in gastric carcinoma and colon cancer.

These observations suggest that activated Janus kinase 2/signal transducer and activator of transcription (3 JAK2-STAT3) signaling could be a possible mechanism to mediate transformation or de-differentiation of thyroid cancer cells.

However, the biological mechanisms by which BMI in humans may relate to thyroid cancer remain speculative to date. Of these, the most convincing links adiposity excess and the consequent increase in insulin production, which is responsible for tumor growth by increasing free insulin-like growth factor (IGF)-1, which in turn stimulates cell proliferation and suppresses apoptosis and has been positively linked to thyroid cancer (44).

Although hyperinsulinemia *per se* has not been directly linked to thyroid carcinogenesis, hyperglycemia has been linked to thyroid cancer by some studies (45).

Furthermore, adiposity may increase the risk of thyroid cancer through its effects on thyroid-stimulating hormone (TSH) (46), which is an independent predictor of thyroid malignancy (increased visceral fat correlates with increased serum fT3, a possible expression of adaptive thermogenic phenomena, and TSH with likely altered negative feedback from circulating thyroid hormones).

BMI also correlates inversely with circulating adiponectin levels, and thyroid tumors express receptors for adiponectin. However, in the absence of a major direct effect of adiponectin on thyroid cancer cell lines *in vitro*, the negative association could be attributed to indirect effects of adiponectin possibly through metabolic regulation and insulin resistance (47).

Stansifer et al. (48), in a 2014 retrospective study considering 467 patients with thyroid tumors (404 papillary, 47 follicular, 13 medullary, and 3 anaplastic tumors) and 255 controls, show no positive correlation between obesity and thyroid cancer risk.

Other studies found a correlation between a high BMI and a more advanced stage, at the time of diagnosis, of thyroid cancer and also with a more aggressive histopathological subtype (49, 50), but not all studies agree that this actually increases the risk of developing thyroid cancer (51, 52). In any case, this study considers thyroid tumors in general, including 3 anaplastic ones, so statistically not significant for ATC (only 3 cases).

Schmid et al. (53), in a 2015 review, show a statistically significant 25% higher risk of thyroid cancer in overweight individuals and a 55% higher risk of thyroid cancer in obese individuals compared to their normal-weight peers. When assessed by histological type, obesity was significantly positively correlated with papillary, follicular, and anaplastic thyroid cancers, while it revealed an inverse association with medullary thyroid cancer. Both general adiposity and abdominal adiposity are positively associated with thyroid cancer. However, the relationships with BMI vary significantly depending on the histological type of the tumor (53).

Ma et al. (54), in a 2015 meta-analysis, correlate obesity with a significantly increased risk of thyroid cancer in both men and women, young and old, especially Caucasians and Asians. In a histological subgroup analysis, an increased risk of papillary, follicular, and anaplastic thyroid cancer was observed. However, obesity was associated with a reduced risk of medullary thyroid cancer. As this meta-analysis was considering only obesity, the possibility that the observed associations may be confounded by other lifestyle factors, such as reduced physical activity or dietary factors, cannot be excluded (54).

Leitzmann et al., (41) in a 2010 prospective study, show a weak and inconsistent positive association between BMI and thyroid cancer risk, as weak and inconsistent is the association with certain hormonal and reproductive factors among women, such as advanced age at menarche, advanced age at first delivery, a history of miscarriage of the first pregnancy, and use of oral contraceptives. In contrast, dietary intake of fish and cruciferous vegetables and cigarette smoking was inversely related to thyroid cancer (41).

Despite the fact that numerous studies in the past have made strong indications for multinodular goiter surgery, the current trend is still linked to obvious compressive symptoms, hyperfunction, or a real suspicion of cancer (41, 55).

The studies included in this review are summarized in **Table 3**.

**Table 3:** Summary of studies included.

	Authors	Study	Year	No. of patients
1.	Maatouk, J.,et al. (19)	Case report	2009	1
2.	Chandrakanth, A., et al. (22)	Review	2006	1,556
3.	Zivaljevic, V., et al. (13)	Case Control Study	2004	110
4.	Zivaljevic, V., et al. (13)	Case Control Study	2008	126
5.	Kim, W. G., et al.	Epidemiologic Study	2013	Animal and Laboratory studies
6.	Leitzmann, M. F., et al. (41)	Experimental study	2010	3,490,300
7.	Zivaljevic,V., et al. (13)	Case Control Study	2014	126
8.	Mitsiades, N., et al. (47)	Epidemiologic/Case Control Study	2011	175
9.	Stansifer, K. J., et al. (48)	Retrospective Study	2015	467
10.	Paunovic, I. R., et al. (3)	Retrodpective Study	2015	150
11.	Zivaljevic, V., et al. (13)	Case Control Study	2010	126
12.	Kitahara, C.M., et al. (52)	Risk Assessment Study	2012	197,710
13.	Engeland, A., et al. (42)	Case Control Study	2006	3,046
14.	Apostolou, K., et al. (59)	Observational Study	2021	3,233
15.	Ma, J., et al. (54)	Metanalysis	2015	12,620,676

Considering the scarcity of bibliographic data, the studies include a heterogeneous literature (retrospective, epidemiological, experimental, case control and review studies).

Considering the scarcity of bibliographic data, the studies include a heterogeneous literature (retrospective, epidemiological, experimental, case–control, and review studies).

Finally, further studies are needed to clarify the potential biological mechanisms underlying the possible positive relationship between adiposity and thyroid cancer risk; currently, no specific correlation has emerged with ATC.

The persisting uncertainties about the very nature of this disease are among the causes of a difficult therapeutic approach, which to this day remains largely unsuccessful, given the extreme severity of the prognosis in spite of aggressive and combined treatments, as suggested in literature (56). Better therapeutic prospects may come, in the near future, from the identification of target therapies, which are still under study (57) since surgical resection does not improve survival in cases of advanced anaplastic cancer (58). Multinodular thyroid disease is associated with a significant rate of incidental thyroid cancer, sometimes underestimated. For this reason it is necessary to know other risk factors to assess the malignant potential of a MNG estimated as benign (59).

## Conclusion

The low incidence and poor prognosis within a few months of diagnosis negatively influence the finding of specific risk factors for ATC: rarity and exit within a few months of diagnosis compromise the collection of specific data.

The identification of independent or additional risk factors in the anaplastic transformation of a DTC and the understanding of the pathways of anaplastic transformation could help in the development of strategies for the early diagnosis and treatment of ATC.

A more aggressive surgical attitude in case of DTC could prevent the anaplastic transformation of the 20%–25% of ATC cases arising on diagnosable DTC. But is an aggressive approach in 98% of patients with DTC conceivable to prevent less than 2% of anaplastic thyroid cancers?

There are few epidemiological studies on this type of cancer.

A long observation period is needed to collect data on a few cases, given their rarity, in order to implement prevention and early detection strategies.

Therefore, it is particularly important to obtain epidemiological information on ATC on a large scale, from large databases, through multicenter studies involving high-volume centers worldwide.

## Data Availability Statement

The raw data supporting the conclusions of this article will be made available by the authors without undue reservation.

## Ethics Statement

Ethical review and approval were not required for the study on human participants in accordance with the local legislation and institutional requirements. Written informed consent for participation was not required for this study in accordance with the national legislation and the institutional requirements.

## Author Contributions

Each author made substantial contributions to the work, approved the submitted version, and agreed to be personally accountable for the author’s own contributions and for ensuring that questions related to the accuracy or integrity of any part of the work are appropriately investigated, resolved, and documented in the literature. Conceptualization: GG and GSc. Methodology: GSa. Validation: GO. Formal analysis: PR, AC, and SM. Investigation: MP, SC, and FS. Resources: RT and GM. Data curation: GO and GM. Writing: GSc and GSa. Original draft preparation: GS. Statistical analysis: SM. Writing—Review and editing: GSc. Visualization: GO and GG. Supervision: GSc. All authors contributed to the article and approved the submitted version.

## Conflict of Interest

The authors declare that the research was conducted in the absence of any commercial or financial relationships that could be construed as a potential conflict of interest.

## Publisher’s Note

All claims expressed in this article are solely those of the authors and do not necessarily represent those of their affiliated organizations, or those of the publisher, the editors and the reviewers. Any product that may be evaluated in this article, or claim that may be made by its manufacturer, is not guaranteed or endorsed by the publisher.
